# Selenium and Iodine as Susceptibility Modifiers of Thyroid Disruption Associated with Metals: An Overview of Human and Experimental Research Data

**DOI:** 10.3390/jox16050164

**Published:** 2026-08-31

**Authors:** Maria-Nefeli Georgaki, Despoina Ioannou, Kanellos Skourtsidis, Georgios Kiosis, Theodora Papamitsou, Dimosthenis Sarigiannis

**Affiliations:** 1 Department of Environmental Engineering, Aristotle University of Thessaloniki, University Campus, 54124 Thessaloniki, Greece; sarigiannis@eie.gr; 2 HERACLES Environmental Health Research Group, 6th km Charilaou–Thermi Road, P.O. Box 60361, Thermi, 57001 Thessaloniki, Greece; 3 Laboratory of Histology and Embryology, School of Medicine, Aristotle University of Thessaloniki, University Campus, 54124 Thessaloniki, Greece; dioana@auth.gr (D.I.); kskour@auth.gr (K.S.); gkiosis@auth.gr (G.K.); thpapami@auth.gr (T.P.); 4 National Hellenic Research Foundation, 48 Vassileos Constantinou Ave., 11635 Athens, Greece; 5 Environmental Health Engineering, School for Advanced Study IUSS Pavia, Palazzo del Broletto, Piazza della Vittoria 15, 27100 Pavia, Italy

**Keywords:** iodine, selenium, thyroid hormones, endocrine disruption, adverse outcome pathway, human biomonitoring

## Abstract

**Background:** Thyroid hormone synthesis, deiodination, and redox regulation depend on iodine availability and selenium-dependent proteins. Therefore, these nutrients may alter sensitivity to thyroid disturbance brought on by metals and metalloids; nevertheless, direct human evidence has not been compiled independently from rescue experiments. **Goal**: To determine the mechanistic, biomarker, and study-design needs for interpretable human research, as well as to critically assess whether iodine or selenium alters metal-associated thyroid effects. **Methods:** Terms for metals, metalloids, iodine, selenium, and thyroid endpoints were used to search PubMed/MEDLINE until 14 July 2026. The database search was improved by selective forward citation searching, backward citation searching, and exact-title and DOI retrieval. A thyroid-specific outcome, a measurable or experimentally manipulated iodine or selenium variable, and a metal or metalloid exposure were all necessary for studies to be eligible. An author-developed framework that distinguished between formal interaction, stratification, joint-exposure modeling, contextual co-measurement, factorial nutritional-status experiments, physiologically interpretable supplementation, and pharmacological or nanoparticle rescue was used to categorize experimental evidence from humans and mammals. **Results:** Seven human studies and nine mammalian experimental studies made up the core evidence set. One additional human study was retained as contextual evidence. The results of the three human studies that directly assessed modification were mixed. One showed no clear interactions with iodine or selenium, one discovered an isolated strontium-by-iodine interaction, and one reported a suggestive mercury-by-iodine-supplement interaction. Most experimental trials employed high-dose, combination, parenteral, or nanoparticle rescue methods, but they more consistently demonstrated mitigation of thyroid damage by selenium-containing treatments. **Conclusions:** Although iodine- and selenium-dependent sensitivity is biologically feasible, there is currently little human data to support a consistent protective or detrimental modifying impact. Rather than supporting population-level prevention, experimental rescue promotes mechanistic modifiability. Repeated iodine testing, functional selenium biomarkers, metal speciation, vulnerable-window sampling, thyroid-specific outcomes, and predetermined interaction analyses are all necessary for future research.

## 1. Introduction

Iodine (I) and selenium (Se) are essential to thyroid hormone physiology. Iodide is the substrate for the synthesis of thyroxine (T4) and triiodothyronine (T3), whereas Se is incorporated into iodothyronine deiodinases, glutathione peroxidases, thioredoxin reductases, and other selenoproteins that regulate hormone activation and inactivation, peroxide handling, and cellular redox homeostasis [1,2,3]. Because thyroid hormone synthesis requires controlled hydrogen peroxide generation, physiological hormone production and oxidative injury are separated by a narrow regulatory margin [3,4].

There is no straightforward more-is-better link between iodine and selenium. Thyroid physiology can be disrupted by both excess and deficiency, and it is impossible to accurately determine status without taking matrix, timing, biological range, and demographic context into account. Although it has significant within-person variability and is inadequate for categorizing long-term individual status, a single spot urine iodine concentration is helpful for describing population distributions [5,6]. Whole-blood selenium integrates a longer period; serum or plasma selenium mostly reflects relatively recent exposure, and functional markers like glutathione peroxidase activity and selenoprotein P react differently over the intake range [7].

Through non-equivalent mechanisms, such as interference with sodium iodide symporter (NIS)-mediated uptake or thyroid peroxidase (TPO)-dependent organification, thyroidal oxidative stress, altered deiodination, modifications in hormone transport, displacement of essential elements, and disruption of hypothalamic–pituitary–thyroid (HPT) feedback, metals and metalloids can disrupt the thyroid system. Broad reviews do not support a single consistent metal-thyroid effect because epidemiological findings vary across elements, species or chemical forms, biological matrices, life stages, thyroid endpoints, and statistical models [4,8,9]. According to Ankley and Edwards (2018) [10], the adverse outcome pathway (AOP) framework offers an organized manner to arrange mechanistic evidence from a molecular initiating event (MIE) to an adverse outcome (AO) via connected key events (KEs) and key-event relationships (KERs). Hormone synthesis disruptions, NIS and TPO function, circulating hormone concentrations, follicular-cell responses, and downstream developmental or organ-level effects are examples of thyroid-related AOP networks [11,12]. Iodine and selenium are not considered universal MIEs in this review. Rather, they are seen as possible regulating factors that could change the amount or direction of particular KEs, including iodine’s substrate availability and organification and selenium’s deiodination and selenoprotein-mediated redox regulation.

Iodine and selenium are thus probable susceptibility modifiers. Limited iodine availability may exacerbate the functional repercussions of interference with iodide uptake or hormone production, whereas a high or rapid iodine load can impede organification or hormone release in vulnerable thyroids. Thyroidal peroxide detoxification, T4-to-T3 conversion, and the reaction to metal-associated oxidative damage can all be impacted by selenium levels. Arbitrary dichotomization may obscure, magnify, or reverse an apparent modifier impact; these effects are unlikely to be linear across the deficiency–adequacy–excess continuum [1,2,3].

The literature is also particularly susceptible to analytical overinterpretation. Regression model adjustments for iodine or selenium do not show effect modification. A mixture model is only useful when it is possible to interpret the nutrient’s contribution or non-additive role. Similarly, pharmacological rescue may be demonstrated by a high-dose selenium nanoparticle given during toxicant exposure, but this does not prove that baseline selenium status alters risk at nutritionally relevant human exposures. Therefore, it is necessary to distinguish between formal interaction, subgroup comparisons, joint-exposure modeling, and rescue studies. Additionally, subgroup significance by itself should not be construed as interaction [13].

To the best of our knowledge, this is the first modifier-centered critical synthesis to require the simultaneous presence of a metal or metalloid exposure, a measured or experimentally manipulated I or Se variable, and a thyroid-specific endpoint. Therefore, the main objective was to evaluate thoroughly whether I or Se alters metal-associated thyroid effects in evidence from humans and mammals. Identifying a formal effect modification from stratified, joint-exposure, contextual co-measurement, factorial nutritional-status, supplementation, and pharmacological-rescue designs; integrating directly measured thyroid outcomes within an AOP-informed framework while separating observed from inferred relationships; and identifying the biomarker and study-design requirements for future human biomonitoring research were the secondary objectives.

## 2. Methods

### 2.1. Review Design

**Figure 1:**
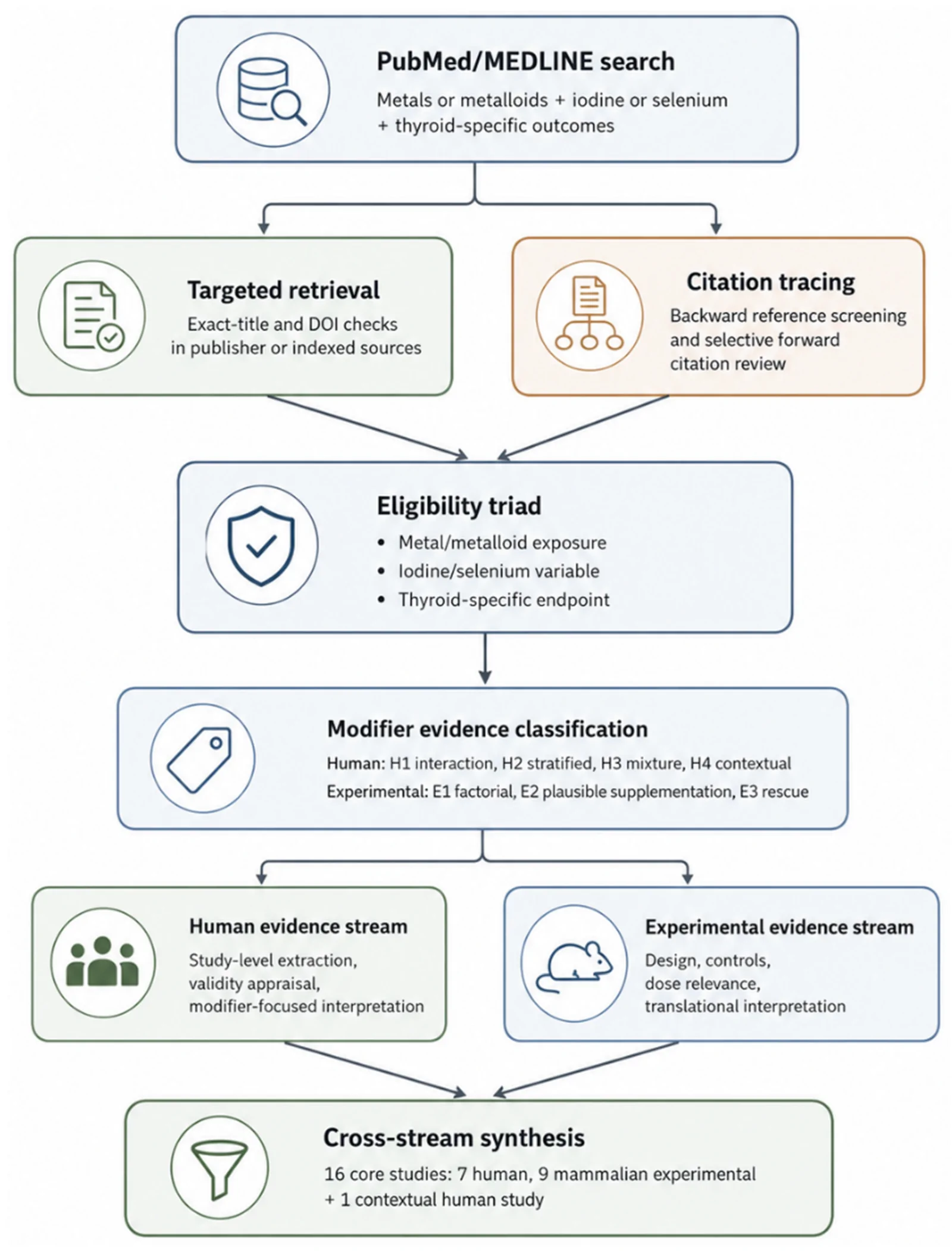
Workflow for identifying, qualifying, classifying, and synthesizing evidence. The PubMed/MEDLINE search, targeted retrieval, citation tracking, required eligibility trio, modifier-evidence classification, and distinct human and experimental synthesis are all summarized in the diagram. This is a methodical process rather than a PRISMA flow diagram since the initial record.

This review was structured as a critical evidence synthesis, with concise evidence identification, specific eligibility criteria, study-level verification, and AOP-informed interpretation. It is not classified as a comprehensive systematic review because the study did not employ a registered protocol, double independent screening, or a thorough multi-database search. This stricter categorization avoids implying procedures that were not carried out and are methodologically intentional. Evidence identification was completed and updated on 14 July 2026. Using controlled vocabulary and title/abstract phrases related to three concepts, metals and metalloids, iodine and selenium, and the thyroid system, PubMed/MEDLINE was searched from the beginning of the database. Selective forward citation searching, backward reference-list review of eligible studies and pertinent reviews, and exact-title and DOI retrieval from publisher repositories were used in addition to database searching. There was no constraint on the date. Figure 1 summarizes the process for identifying, qualifying, and synthesizing evidence.

Core PubMed/MEDLINE strategy: ((“Metals”[Mesh] OR “Heavy Metals”[Mesh] OR metal*[tiab] OR metalloid*[tiab] OR chromium[tiab] OR chromate*[tiab] OR “hexavalent chromium”[tiab] OR cadmium[tiab] OR lead[tiab] OR mercury[tiab] OR methylmercury[tiab] OR arsenic[tiab] OR antimony[tiab] OR nickel[tiab] OR cobalt[tiab] OR manganese[tiab] OR copper[tiab] OR zinc[tiab] OR iron[tiab] OR molybdenum[tiab] OR vanadium[tiab] OR strontium[tiab]) AND (“Iodine”[Mesh] OR “Selenium”[Mesh] OR iodine[tiab] OR iodide[tiab] OR “urinary iodine”[tiab] OR “iodine supplement*”[tiab] OR selenium[tiab] OR selenoprotein*[tiab] OR “selenoprotein P”[tiab] OR “glutathione peroxidase”[tiab] OR selenomethionine[tiab] OR selenite[tiab] OR “selenium nanoparticle*”[tiab]) AND (“Thyroid Gland”[Mesh] OR “Thyroid Hormones”[Mesh] OR “Thyroid Diseases”[Mesh] OR thyroid*[tiab] OR thyroxine[tiab] OR triiodothyronine[tiab] OR TSH[tiab] OR thyroglobulin[tiab] OR thyroperoxidase[tiab] OR “thyroid peroxidase”[tiab] OR deiodinase*[tiab] OR “sodium iodide symporter”[tiab] OR hypothyroid*[tiab] OR hyperthyroid*[tiab] OR goiter[tiab] OR goitre[tiab])). Modifier terms were used in supplementary searches but were not mandatory because relevant studies frequently omit interaction terminology from titles and abstracts.

### 2.2. Inclusion and Exclusion Criteria

Eligible primary studies had to follow a three-part rule: (1) a measured or experimentally administered metal or metalloid exposure; (2) a measured or experimentally modified iodine or selenium variable; and (3) at least one thyroid-specific outcome. Mammalian in vivo experiments and human observational or interventional investigations were among the eligible designs. TSH, total or free T4/T3, reverse T3, thyroglobulin, thyroid antibodies, defined thyroid disease, gland morphology or histopathology, iodide uptake, NIS or TPO function, thyroglobulin synthesis, deiodinases, thyroid-localized redox markers, and other obviously thyroid-related molecular or functional measurements were among the thyroid-specific endpoints.

Studies were removed from the core modifier evidence set if iodine or selenium appeared only in a baseline table or as a generic covariate with no interpretable joint or modifying role. These studies were only retained if they provided significant contextual co-measurement. Results of general systemic oxidative stress without a thyroid-specific test were not included. Nutritional studies without an environmental or toxicological metal context, radioiodine therapy, and studies of iodine or selenium as the only exposure were not included. Only when assessed in an environmental, occupational, toxicological, or combination context might essential elements including copper, zinc, iron, manganese, and selenium be considered exposure factors.

### 2.3. Analytical Identification and Analysis of Modifier Evidence

The roles that iodine and selenium were allocated varied significantly among the eligible research. Iodine or selenium was assessed in some studies as a possible moderator of the relationship between metal exposure and a thyroid outcome; in other studies, it was added to a joint-exposure model, included solely as an adjustment variable, or used in experiments as a therapeutic or nutritional intervention. An author-developed classification framework was used to prevent these analytically different designs from being regarded as similar indications of vulnerability (Table 1).

The framework was used to describe the analytical or experimental association between the metal exposure, the iodine or selenium variable, and the thyroid-specific endpoint. It was established before the final cross-study interpretation. Rather than on the direction, quantity, or statistical significance of the results, the classification was based on the study design and analysis described. Every study was given a single primary category; if a study included more than one pertinent analytical or experimental component, a secondary description was added.

This approach is neither a numerical quality scale, a certainty-of-evidence grading system, nor a verified risk-of-bias tool. Therefore, it is not appropriate to interpret the categories as an ordinal rating of the overall quality of the study. Rather, they show how directly a study design can answer the modifier-centered review issue. As outlined in Section 2.4, methodological validity was assessed independently for exposure measurement, modifier assessment, temporal alignment, confounding, statistical power, selective reporting, dose relevance, and external validity.

An explicitly reported interaction term, product term, response-surface interaction, or comparable model-based estimate on a specified statistical scale, along with an estimate of uncertainty or a formal test of heterogeneity, were necessary for formal effect modification in human investigations. Stratum-specific correlations were categorized independently in the absence of a systematic comparison between strata. There was no indication that the subgroup effects were different if one subgroup had a statistically significant association and another had a non-significant association. Because additive and multiplicative interaction address distinct scientific topics and are not interchangeable, interaction results were interpreted in accordance with the scale on which they were calculated [13].

Supplementation and therapeutic rescue were separated from factorial manipulation of iodine or selenium status for experimental investigations. The most straightforward experimental design for determining nutritional susceptibility is a factorial experiment, which examines the reaction to the same metal dose under controlled deficiency, adequacy, or excess circumstances. On the other hand, administering selenium or iodine during or after metal exposure mainly assesses whether an intervention reduces toxicity. These studies may reveal sensitive pathways or bolster biological plausibility, but they may not prove that risk is altered by baseline nutritional status. Therefore, rescue data were categorized and evaluated independently from nutritionally reasonable susceptibility designs for high-dose, parenteral, nanoparticle, combined-intervention, or post-exposure treatments.

The following interpretation guidelines were used to apply the categories. First, methodological effectiveness was not equated with the modifier design’s directness. Even if only the former explicitly examined effect modification, a well-conducted H2 study may be more reliable than an H1 study with significant exposure misclassification, insufficient power, or selective interaction testing. In a similar vein, an E1 experiment offers a more direct assessment of nutritional susceptibility than an E3 rescue experiment; nevertheless, its applicability to humans still depends on the model, thyroid endpoints, exposure dose, and nutritional treatment. Second, no study was upgraded based only on statistical significance, biological plausibility, or positive direction of effect. On the other hand, a non-significant interaction test did not always indicate that there had been no change. Only when the interaction estimate was accurate, the modifier and exposure measures were suitable, and the study had adequate power to identify a physiologically significant difference were null results deemed instructive. Third, variables related to iodine or selenium were interpreted in accordance with their true meanings. Supplement use was not considered a direct biomarker of nutritional status, but rather an intake proxy. Total serum or plasma selenium was not thought to reflect functional selenoprotein activity, and a single spot urine iodine measurement was thought to be susceptible to individual-level misinterpretation. The constraints were considered in the independent validity assessment, although studies that used such measures were nonetheless eligible. Fourth, the interpretation of mixture models as interaction analyses was not automatic. Only when the model explicitly evaluated non-additivity or variation in the metal effect across iodine or selenium levels were studies classified as evidence of formal modification. Studies were assigned to H3 when iodine or selenium contributed to an interpretable joint-exposure or combined result. Fifth, unless the independent effect of each micronutrient could be distinguished, combined interventions were not exclusively assigned to iodine or selenium. Unless the study design provided the controls necessary to determine the impact of each component, experiments combining selenium with zinc, myo-inositol, antioxidants, or other agents were categorized as E3 with a co-intervention descriptor. Finally, H4 studies were used only to characterize the larger measurement context and were not included in the core modifier conclusions. Cross-stream results were only reached after considering design directness, methodological validity, exposure relevance, biological coherence, and applicability to human environmental exposure. Human and experimental categories were synthesized independently.

### 2.4. Mechanistic Mapping Informed by AOP

**Figure 2:**
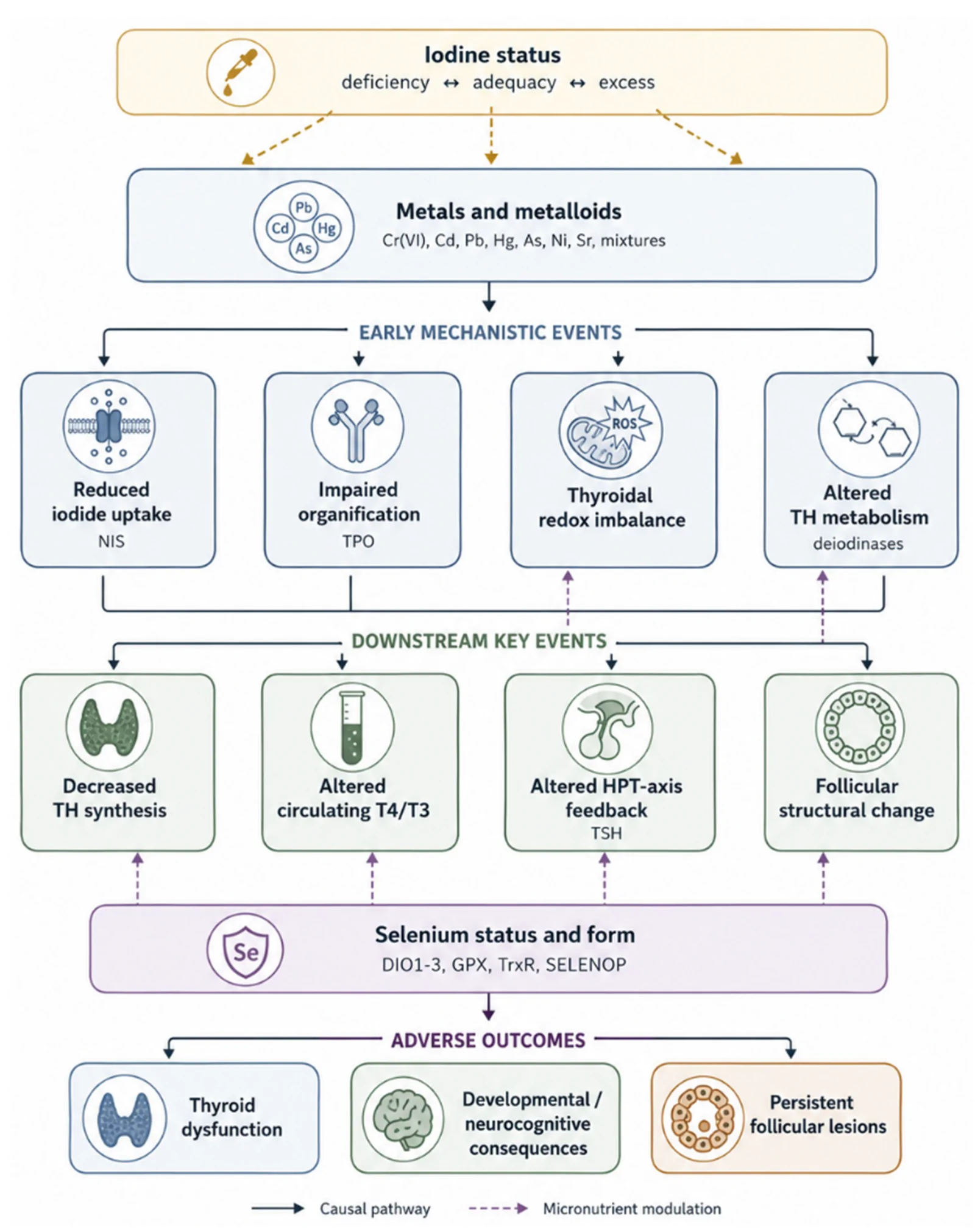
AOP-informed framework for modifying metal-associated thyroid disturbance in a manner dependent on iodine and selenium. Dashed arrows show suggested micronutrient-dependent modulation, while solid arrows show AOP-consistent progression. The included studies and thyroid AOP literature [3,4,11,12] provided information for the nodes and associations. The figure does not represent a recently approved or verified AOP; rather, it is interpretative.

Results were mapped to thyroid-related AOP ideas outlined in mechanistic reviews and mammalian AOP networks to integrate different thyroid endpoints without implying mechanistic equivalency [4,11,12]. Reduced NIS-mediated iodide uptake, poor TPO-dependent organification, thyroidal redox imbalance, and modified deiodinase function were among the potential upstream events. Reduced thyroid hormone synthesis, changed tissue or circulatory hormone availability, disrupted HPT-axis feedback, follicular structural alteration, and clinically or developmentally significant effects were among the downstream events. Iodine and selenium were depicted as modifying factors rather than universal MIEs: iodine status may affect substrate availability and organification, whereas selenium status and chemical form may influence deiodination and selenoprotein-mediated redox defense. Only events directly measured in an included study were considered observations, while linkages supported only by external mechanistic literature were classified as inferred. This process does not constitute formal AOP formulation, endorsement, or weight-of-evidence review; rather, it is an interpretive mapping informed by AOP. Figure 2 shows the final framework.

### 2.5. Data Extraction, Evaluation of Validity, and Synthesis

The design, population or animal model, sample size if available, metal species and identity, exposure matrix or dose, iodine or selenium measure or intervention, timing, thyroid endpoint, statistical or experimental modifier design, direction and magnitude of the primary findings, and the principal validity constraint were all documented for each study. Only when the main study’s abstract or entire text could be used to confirm the numerical estimations were they reported.

The validity assessment was domain-based rather than reduced to an unvalidated total score. Selection, exposure and modifier measurement, temporal alignment, thyroid-outcome validity, confounding, multiplicity, interaction scale, precision, and selective subgroup reporting were all evaluated in human evidence. Control structure, nutritional status definition, toxicant and selenium dose relevance, randomization/blinding reporting, thyroid specificity, co-interventions, and human application were all considered when evaluating experimental findings. A single spot urine iodine sample was regarded as weak for individual long-term classification; supplement use was not considered a biomarker of iodine status; computed homeostasis indices were not equated with direct enzyme assays; and total chromium was not considered Cr(VI)-specific unless speciation was carried out.

Prior to cross-stream interpretation, human and experimental findings were combined independently. Because the designs, exposure species, nutritional factors, outcome scales, and modifier models were not sufficiently comparable, no pooled impact estimate was computed. The direction was classified as null, mixed, attenuation, amplification, or undetermined; however, statistical significance vote counting was not employed to demonstrate an overall effect.

## 3. Results

### 3.1. Evidence Distribution

Sixteen important studies, seven involving humans and nine involving animals, made up the final evidence set. Another human study that looked at thyroid outcomes, mercury exposure, blood selenium, and urine iodine but did not evaluate modification was retained as H4 contextual evidence. The identification and classification process is summarized in Figure 1; the features and findings of human research are shown in Table 2; and the experimental evidence is shown in Table 3. Only three human studies explicitly evaluated iodine- or selenium-dependent alteration using formal or stratified analysis (H1/H2). Four more human trials provided joint-exposure or mixture data (H3); however, their ability to differentiate dietary susceptibility was limited. Three of the nine experimental trials most closely mirrored factorial or status-oriented designs (E1/E2), while six were mostly rescue studies (E3), often using selenium nanoparticles or selenium in combination with zinc or myo-inositol. The distribution itself is instructive: direct evaluations of baseline human vulnerability are significantly less prevalent than intervention-based rescue after toxicant challenge.

### 3.2. Human Evidence

Table 2 summarizes the human evidence. The table shows the major validity restriction for each discovery and separates formal interaction and stratification evidence from joint-exposure modeling and contextual co-measurement. Iodine or selenium measurements did not always reflect the biologically relevant exposure window, estimates were frequently underpowered, and direct modifier evidence was scarce across investigations.

**Table 2:** Human evidence on joint exposures, contextual measurements, and iodine or selenium as potential modifiers.

Study/Category	Population and Design	Exposure and Modifier Assessment	Verified Thyroid-Related Findings	Interpretation and Principal Limitation
Meltzer et al., 2002 [14] H3	32 women; 15-week fish-intake and selenium-supplement intervention.	Fish intake; selenium methionine or selenite 400 micrograms/day; placebo group; blood As and Se.	In the non-selenium group, final blood As correlated positively with T4:T3 (r = 0.80, *p* < 0.02; *n* = 8). Initial inverse As-T3/T4 correlations disappeared with selenium supplementation.	Suggestive Se-As interaction, but very small groups, high selenium intake, correlation-based analysis, and predominantly organic seafood arsenic.
Llop et al., 2015 [15] H1/H2	1407 pregnant women, Spanish INMA cohort.	Cord-blood total Hg; self-reported iodine-supplement use; maternal thyroid hormones at approximately 13 weeks.	Hg-TT3 association was stronger among iodine-supplement users (beta = −0.08; 95% CI −0.15 to −0.02) than non-users; interaction *p* = 0.07. Doubling Hg corresponded to an estimated 5.5% lower TT3 among users.	Suggestive amplification, not definitive interaction. Supplement use is an imperfect iodine-status proxy and cord Hg was measured months after the thyroid outcome.
Jain and Choi, 2016 [16] H3	US NHANES 2011–2012 adults; sex-stratified cross-sectional analysis.	Blood Mn, Se, Cd, Pb, Hg; serum Fe, Zn, Cu; multi-element interaction modeling.	Element-thyroid associations varied by sex and co-element adjustment. No consistent selenium-mediated attenuation of toxic-metal associations emerged.	Joint-exposure evidence only. Many parameters and interactions, cross-sectional design, and limited biological interpretability of individual interaction terms.
Molin et al., 2017 [17] H3	38 healthy volunteers; 14-day randomized seafood-feeding trial.	Cod, salmon, blue mussel, or potato control; plasma total As and Se; urinary iodine.	Seafood increased TSH, with plasma As a dominant predictor. Selenium entered models inversely and iodine positively, but formal nutrient-by-As modification was not established.	Randomized exposure strengthens temporality, but seafood simultaneously changes As species, iodine, selenium, and other nutrients; total As is dominated by organic species.
Gustin et al., 2021 [18] H1	Approximately 550 pregnant women in a Swedish birth cohort.	Urinary Cd and iodine; erythrocyte Cd, Pb, Hg; plasma selenium; thyroid hormones.	Cd was positively associated with total T4/T3 and free T3; Hg was inversely associated with free and total T3 and the fT3:fT4 ratio. BKMR showed no clear metal-iodine or metal-selenium interactions; Hg x Se interaction *p* > 0.10.	Most informative direct human analysis and essentially null for modification. Residual timing and measurement limitations remain, but iodine and selenium varied widely.
Hu et al., 2021 [19] H3	329 rural adults along the Yangtze River; cross-sectional mixture study.	Eight plasma PBDEs and 14 urinary metals, including urinary selenium.	FT4 was inversely associated with urinary Sr and positively associated with urinary Se; FT3 was inversely associated with urinary As. BKMR did not identify robust pairwise interactions.	No consistent modifier signal. Urinary selenium is not an ideal long-term selenium-status biomarker, and PBDE co-exposure complicates metal-specific inference.
Ge et al., 2023 [20] H1	328 occupationally exposed workers; cross-sectional study.	Twenty-two blood-cell metals; spot urinary iodine; TSH, T3/T4 and calculated SPINA-GD/GT.	One iodine-dependent interaction was reported for Sr and calculated peripheral deiodinase activity (*p* interaction = 0.026); the association was positive in iodine-insufficient participants and reversed in the adequate group. No modification was found for the other metals.	Isolated positive interaction requiring replication. Single spot iodine measure, multiple testing, cross-sectional design, and calculated rather than directly measured deiodinase activity.
Correia et al., 2020 [21] H4 context	55 formerly Hg-exposed men and 55 matched controls, approximately 14 years after exposure.	Urinary Hg and iodine; serum selenium; thyroid hormones, antibodies, and ultrasonography.	Formerly exposed men had higher TSH and more TSH values above 4.2 mIU/L, plus more echogenicity alterations. Iodine and selenium were measured but not tested as modifiers.	Important contextual co-measurement, but not evidence that iodine or selenium modified the Hg-thyroid association.

As, arsenic; BKMR, Bayesian kernel machine regression; Cd, cadmium; CI, confidence interval; Cu, copper; Fe, iron; fT3, free triiodothyronine; fT4, free thyroxine; H1–H4, human modifier-evidence categories; Hg, mercury; INMA, INfancia y Medio Ambiente; Mn, manganese; NHANES, National Health and Nutrition Examination Survey; Pb, lead; PBDEs, polybrominated diphenyl ethers; Se, selenium; SPINA-GD, calculated peripheral deiodinase activity; SPINA-GT, calculated thyroid secretory capacity; Sr, strontium; T3, triiodothyronine; T4, thyroxine; TSH, thyroid-stimulating hormone; TT3, total triiodothyronine; Zn, zinc.

Table 2 summarizes the characteristics and conclusions of human research, separating formal interaction and stratification evidence from joint-exposure modeling and contextual co-measurement. Across investigations, I or Se measurements did not always correspond to the physiologically relevant exposure window, interaction estimates were usually underpowered, and direct modifier evidence was limited.

#### 3.2.1. Iodine as a Modulator of Human Susceptibility

Gustin [18] offered a comprehensive null interaction analysis, but the direct human iodine evidence was restricted to the Llop and Ge [20] investigations. Women who reported using iodine supplements showed a greater inverse mercury-TT3 connection, according to Llop et al., 2025 [15]. However, the formal interaction did not exceed the traditional 0.05 threshold, and supplement use did not prove biological iodine status, deficit, adequacy, or dose adherence. Another issue with the temporal sequence was that while mercury was found in cord blood at delivery, thyroid hormones were evaluated in the early stages of pregnancy. Therefore, rather than being confirmatory, the result is hypothesis-generating.

Ge et al. [20] determined peripheral deiodinase activity and discovered a statistically significant iodine interaction for strontium, but they found no comparable change for the other metals. The solitary interaction may have a context-specific impact or a false positive result because urinary iodine was based on a single spot sample, the population was occupationally selected, the study was cross-sectional, and numerous exposure-outcome combinations were explored. Using urine iodine and combination techniques throughout pregnancy, Gustin et al. [18] discovered no discernible interaction. Iodine deficiency or supplementation did not consistently increase or decrease metal-associated thyroid alterations in any of the three investigations.

#### 3.2.2. Selenium as a Modulator of Human Susceptibility

The modest Meltzer intervention provided the strongest suggestion of selenium-related alteration. Although there were only eight women in each group, the dose was 400 micrograms per day, and seafood arsenic was not comparable to long-term exposure to inorganic arsenic, selenium supplementation changed relationships between blood arsenic and circulating thyroid hormones. The outcome is not very direct for evaluating environmental danger, but it does support biological interaction.

Plasma selenium did not significantly alter metal-thyroid relationships in Gustin et al. [18] The Jain and Choi [16] analysis did not produce a stable, clinically interpretable selenium-protection pattern, but it did demonstrate that estimations for hazardous and essential elements relied on sex and co-element modeling. Hu et al. discovered no strong mixture interaction but a favorable urine selenium-FT4 connection. Although iodine, selenium, arsenic species, and the food matrix co-varied, Molin et al. [17] found that selenium was a negative predictor in models of seafood-related thyroid alteration. In general, a prespecified, sufficiently powered test of selenium change was less prevalent than co-measurement and joint modeling.

### 3.3. Experimental Evidence

Nine studies on mammals have assessed the effects of iodine or selenium on metal-associated thyroid outcomes (Table 3). Six studies mainly assessed rescue following a high-dose toxicant challenge, while three approximated nutritional-status or factorial questions. Therefore, the design, dose, route, control structure, and whether the intervention reflected baseline state or pharmacological therapy were taken into consideration when interpreting the experimental evidence.

**Table 3:** Mammalian experimental evidence on iodine- and selenium-dependent modification of metal-associated thyroid effects.

Study/Category	Model and Metal Exposure	Iodine/Selenium Design	Main Thyroid-Relevant Finding	Translational Interpretation
Glattre et al., 1995 [22] E1	78 Wistar rats; arsenate in drinking water for 4 weeks.	Selenite alone, arsenate alone, combined exposure, and controls.	As and Se accumulated in thyroid. Arsenic alone produced obvious thyroid histological toxicity, whereas selenium or combined treatment showed minor or no changes.	Factorial evidence of interaction, but exposure levels were high and reporting predates current toxicology standards.
Miyazaki et al., 2005 [23] E1	Pregnant mice; sodium arsenite during gestation.	Selenium-deficient versus selenium-adequate diets.	Selenium deficiency increased arsenic accumulation by 48% in maternal liver and 31% in fetal brain; fetal-brain DIO2 activity increased four-fold under combined deficiency and arsenic exposure.	Strong susceptibility design, but the key deiodinase endpoint was fetal brain rather than thyroid gland and direct thyroid disease was not demonstrated.
Kotyzová et al., 2005 [24] E1/E2	Female Wistar rats; chronic As(III) and bromine exposure for 8 weeks.	Iodine- and/or selenium-enriched diets.	Iodine and/or selenium altered thyroid element retention and reduced bromine uptake into thyroid to roughly half of unsupplemented values.	Supports element-level interaction, but thyroid hormones and clinical function were not the main outcomes.
Hammouda et al., 2008 [25] E3	30 male Wistar rats; cadmium in drinking water for 35 days.	Selenium, zinc, or combined selenium plus zinc co-treatment.	Cadmium increased relative thyroid weight and TSH and reduced T4. Selenium partially protected T4; combined selenium plus zinc more fully corrected T4, TSH, thyroid weight, and cadmium accumulation.	Positive rescue, but high exposures and the strongest effect required a co-intervention, preventing attribution to selenium alone.
Hassanin et al., 2013 [26] E3	20 male rats; single intraperitoneal Cr(VI) dose.	Selenium nanoparticles 0.5 mg/kg intraperitoneally for 5 days.	Cr(VI) reduced free T3/T4 and glutathione and increased oxidative and histological injury; nano-selenium attenuated hormonal, redox, and tissue changes.	Clear pharmacological rescue with very small groups, parenteral dosing, and no relevance to ordinary selenium status.
Atteia et al., 2018 [27] E3	Male rats; lead acetate exposure over 15 weeks.	Selenium nanoparticles administered concurrently.	Nano-selenium attenuated lead-associated reductions in free T3/T4, TSH elevation, oxidative injury, thyroid lead accumulation, altered deiodinase-1 expression, and miR-224 changes.	Mechanistically rich rescue evidence, but nanoparticle dosing and concurrent treatment preclude nutritional extrapolation.
Benvenga et al., 2020 [28] E3	Mice; cadmium chloride for 14 days.	Selenomethionine alone or with myo-inositol.	Selenium reduced inflammatory chemokines but incompletely restored structure; myo-inositol plus selenium more consistently normalized follicular and inflammatory indices.	Combination benefit cannot be assigned to selenium alone; no circulating thyroid-hormone endpoint was central.
Fedala et al., 2021 [29] E3	30 pregnant Wistar rats; potassium dichromate during early gestation.	Selenium, zinc, or combined treatment.	Cr(VI) induced hypothyroid hormone changes, oxidative stress, DNA damage, and thyroid histopathology. Selenium or zinc mitigated injury; combined treatment was not uniformly superior.	Supports antioxidant/genoprotective rescue in pregnancy, but acute parenteral exposure and co-treatment limit human relevance.
Salah et al., 2022 [30] E3	Pregnant Wistar rats; nickel chloride during early gestation.	Selenium, zinc, or combined treatment.	Nickel reduced T3/T4, increased TSH, and produced oxidative and histological thyroid injury; selenium and/or zinc attenuated these changes.	Positive rescue under high-dose gestational exposure; nutritional susceptibility and independence of selenium effects remain uncertain.

As, arsenic; As(III), trivalent arsenic; Cr(VI), hexavalent chromium; DIO2, iodothyronine deiodinase type 2; E1–E3, experimental modifier-evidence categories; miR-224, microRNA-224; Se, selenium; T3, triiodothyronine; T4, thyroxine; TSH, thyroid-stimulating hormone.

The apparent constancy of attenuation is centered in rescue designs, as Table 3 summarizes. This pattern suggests biological and therapeutic modifiability under experimental stress, but it cannot be construed as proof that regular iodine or selenium intake prevents thyroid dysfunction in exposed human populations.

#### 3.3.1. Factorial Nutritional Status Evidence

Glattre et al. [22], Miyazaki et al. [23], and Kotyzová et al. [24] were the three experiments that most closely resembled a real susceptibility test. Together, they demonstrated how exposure to arsenic or bromine might affect tissue distribution, histological damage, and deiodinase-related reactions. However, the most instructive deiodinase result was found in the embryonic brain, and only the Miyazaki et al. [23] design specifically contrasted selenium deficiency with sufficiency. Kotyzová et al. [24] was more interested in thyroid element concentrations than in hormone production or illness. Therefore, the entire human pathway from dietary conditions through metal exposure to verified thyroid dysfunction was not replicated by even the strongest experimental susceptibility data.

There is no known mammalian experiment that systematically measured thyroid uptake, TPO activity, thyroidal redox state, deiodinases, circulating hormones, and histopathology while evaluating a deficiency, adequacy, and excess for both iodine and selenium across environmentally realistic metal doses. This is a significant mechanistic gap.

#### 3.3.2. Rescue Evidence

At least one thyroid-specific hormonal, biochemical, molecular, or histological outcome was reduced in all six rescue experiments. Although the apparent consistency is biologically feasible, it should not be confused with proof of vulnerability. Different metals, methods, doses, durations, life phases, selenium forms, and co-interventions were employed in the investigations. In the chromium and lead investigations, selenium nanoparticles were employed; in the cadmium, chromium, and nickel studies, selenium was mixed with zinc; in the cadmium mouse research, it was combined with myo-inositol. Compared to selenium alone, the combination intervention frequently resulted in the most thorough normalization. The idea that selenium-containing therapies can affect redox defense, metal distribution, and thyroid damage under experimental stress is thus supported by the rescue research. It does not prove that supplementation can prevent metal-associated thyroid disease, that low-normal selenium level raises human risk, or that the dosages and formulations employed are safe or effective in people.

### 3.4. Dominant Validity Limitations and a Cross-Stream Evidence Map

Table 4 summarizes the main cross-stream findings. Experimental data demonstrated selenium-responsive rescue under controlled toxicant exposure, while human evidence was strongest for describing uncertainty and weakest for proving reproducible alteration. Misalignment between exposure species, nutritional biomarker, biological window, thyroid endpoint, and analytical claim was the main constraint in both streams. It shows that rather than a uniform protective or detrimental modifying impact, the evidence is more consistent with context-dependent mechanistic influence. Sparse direct testing, inaccurate biomarkers, and limited interaction power are the reasons behind the absence of clinically translatable human findings, which is not proof of no alteration.

### 3.5. AOP-Informed Mechanistic Integration

Four upstream mechanistic domains impaired iodide absorption or organification, thyroidal oxidative damage, altered deiodination, and disruption of HPT-axis feedback were most consistently mapped by the available evidence. More often than direct NIS, TPO, or deiodinase activity, experimental research evaluated oxidative stress, histopathology, and circulating hormones. Most of the human research used homeostasis indices or circulating hormone measurements. As a result, many metal-specific connections to potential MIEs or early KEs are still mechanistically conceivable rather than proven. These areas are integrated into an AOP-informed framework in Figure 2. Selenium status and chemical form are positioned as regulators of deiodination and selenoprotein-mediated redox defense, whereas iodine status is largely positioned as a possible modifier of substrate availability and organification. A complete MIE-to-AO sequence and validated nutritional status were not evaluated in any of the included studies. Therefore, rather than a freshly validated AOP, the synthesis enables the formulation of mechanistically anchored hypotheses.

## 4. Discussion

### 4.1. Principal Findings

The biological plausibility of iodine- and selenium-dependent vulnerability and the direct data available in people differ significantly, according to this important synthesis. The thyroid gland is inherently reliant on selenium-containing proteins for peroxide regulation and thyroid hormone activation or inactivation, as well as on iodine as the substrate for hormone production. However, only three human investigations specifically looked at whether iodine or selenium altered a metal-thyroid connection, and their results did not agree on a consistent direction of action. Although iodine status was not directly evaluated and the formal interaction was only marginally significant, Llop et al., 2025 [15], found a greater inverse mercury-total T3 correlation among iodine supplement users. Ge et al. [20] found one iodine-dependent strontium relationship with a computed deiodinase index, but the other metal interactions were nil. There was no conclusive evidence of metal-by-iodine or metal-by-selenium interaction, according to Gustin et al. [18], who offered the most comprehensive direct pregnancy analysis. When combined, these findings do not prove that iodine or selenium are consistently protective, harmful, or directionally consistent modifiers of thyroid disturbance caused by metals.

The evidence distribution summarized in Table 4 is a significant result. Most human investigations included joint-exposure models where iodine or selenium was one part of a larger mixture, as well as formal or stratified assessments carried out in pregnancy or occupational settings. Most experimental trials were high-dose, concurrent-treatment, nanoparticle, or combined-intervention rescue models, but they more often showed a reduction in thyroid damage following selenium-containing intervention. Therefore, it is not appropriate to interpret the seeming constancy of experimental protection as proof that vulnerability in human populations exposed to the environment is determined by normal iodine or selenium status. The key conclusion is conditional: although the extent, direction, and pertinent biological range of this alteration are still unknown, dietary status may contribute to variation in metal-thyroid connections.

This distinction is essential to the uniqueness of the current review. Different problems are addressed by pharmaceutical rescue research, a factorial nutritional-status experiment, a mixture model, a stratified comparison, a formal interaction analysis, and covariate adjustment. A statistically significant association in one subgroup combined with a non-significant association in another does not show effect modification; additionally, interaction results depend on the scale on which they are estimated; additive and multiplicative interaction are not interchangeable [13]. The review avoids turning biological responsiveness into an unfounded population-level preventive claim by separating these designs prior to synthesis.

### 4.2. Interpretation of Human Evidence

The apparent disagreement across direct human investigations should not be misinterpreted as a basic biological contradiction, as the research operationalized iodine-dependent vulnerability in fundamentally different ways. Gustin et al. [18] measured urinary iodine, Ge et al. classified iodine status using a single spot urine sample, and Llop et al. (2025) [15] used self-reported supplement use. These variables capture three distinct constructs: group-based classification, short-term iodine excretion, and supplement-taking behavior. Adherence, dose, absorption, baseline deficiency, and attained biological state are not established by supplement use. Urinary iodine, on the other hand, is sensitive to recent intake and suitable for characterizing population distributions; however, a single spot measurement is weak for long-term individual classification and susceptible to significant within-person variability [5,6]. Therefore, although all three investigations employed the label of iodine status or exposure, they did not investigate the same modifier construct.

The thyroid results and exposure windows were also not identical. Llop et al. (2025) [15] employed cord-blood mercury collected at delivery to evaluate maternal thyroid hormones early in pregnancy, which creates a temporal-alignment issue unless exposure persistence throughout gestation can be shown. Ge et al. focused on computed SPINA indices instead of direct tissue deiodinase measurements, while Gustin et al. [18] included multiple measured hormone endpoints and metal indicators into a more comprehensive pregnancy analysis. Although calculated homeostasis indices are still model-derived variables and should not be regarded as direct enzyme tests, they may be helpful for system-level interpretation. Furthermore, current clinical and laboratory guidelines emphasize context-specific interpretation and appropriate reference intervals because thyroid tests are sensitive to assay platform, binding-protein changes, gestational timing, medication, acute illness, and thyroid autoimmunity [31,32]. The significance of gestational timing, iodine diet, thyroid autoimmunity, and population-appropriate interpretation of thyroid function tests are also highlighted in the updated American Thyroid Association pregnancy guidelines [33].

Given this setting, Llop et al.’s [15] finding is best viewed as hypothesis-generating rather than established mercury-iodine synergism. In addition to biological interactions, residual confounding by food, supplement indication, socioeconomic characteristics, baseline thyroid susceptibility, or variations in health behavior may be the cause of the greater inverse mercury-total T3 estimate among supplement users. The lack of a direct iodine biomarker and the interaction *p*-value of 0.07 further reduces assurance. Ge et al.’s isolated strontium-by-iodine result is biologically intriguing as well, but it must be repeated because it was derived from several exposure-outcome comparisons, depended on a single spot iodine sample, and included a computed deiodinase index. In contrast, interaction tests are typically underpowered, particularly when exposure and modifier measures contain error; therefore, Gustin et al.’s [18] null interaction analysis is more immediately instructive but should not be taken as evidence that modification is absent. Only when a null estimate is accurate enough to rule out a physiologically significant difference is it persuasive.

Instead of formal susceptibility, co-exposure is mostly informed by the remaining human investigations. Selenium supplementation changed associations between seafood-derived arsenic, selenium, and thyroid-hormone ratios in a modest experiment described by Meltzer et al [14]. The groups were relatively small, the selenium dose was large, and seafood-related organic species dominated the arsenic exposure, even though temporality was stronger than in cross-sectional research. This divergence is toxicologically significant since total arsenic after seafood consumption cannot be interpreted as chronic exposure to inorganic arsenic; arsenic species differ significantly in metabolism and toxicological importance [34]. Randomized dietary allocation helped Molin et al. [17] as well, although seafood affected arsenic species, iodine, selenium, and other nutrients at the same time, making it challenging to identify a modifying influence that was particular to iodine or selenium. These studies do not establish a threshold of nutritional vulnerability, but they do demonstrate that food-based exposures can produce biologically significant joint patterns.

Jain and Choi’s [16] NHANES analysis showed how sex and co-element adjustment affected metal-thyroid estimations, demonstrating how multi-element inference is sensitive to model design. Nevertheless, no consistent pattern of selenium-mediated attenuation was found. Similar element-specific hormone relationships were found by Hu et al. [19] without strong pairwise mixture interactions; in that investigation, urine selenium was also a poor indicator of functional or longer-term selenium status. Although the study did not assess modification, Correia et al. [21] is nevertheless significant because it showed long-lasting thyroid changes following occupational mercury exposure while evaluating urine iodine and serum selenium. Therefore, without proving that either micronutrient changed the mercury–thyroid connection, it adds to the review’s measurement context and plausibility.

Overall, human literature is constrained less by a lack of relevant measurements than by a lack of investigations centered on a predetermined modifier hypothesis. The causal role of iodine and selenium should be established before analysis; repeated or otherwise validated status measures should be obtained, exposure and thyroid sampling should be aligned to the same biological window, the interaction scale should be specified, the interaction coefficient and uncertainty should be reported, and stratum-specific estimates should be provided without solely depending on variations in statistical significance. Because correlated elements, model instability, and multiple testing can result in apparently unique patterns that are difficult to replicate, these conditions are particularly crucial in the study of mixtures.

### 4.3. Analysis of the Experimental Data

The experimental evidence shows that metal-related thyroid damage is biologically changeable, but it does not define nutritionally realistic susceptibility. Due to the manipulation of iodine or selenium conditions in addition to metal exposure, Glattre et al. [22], Miyazaki et al. [23], and Kotyzová et al. [24] most closely addressed a status-oriented inquiry. However, the designs addressed various levels of biological order even within this category. Glattre et al. [22] focused on thyroid histology and the distribution of arsenic and selenium; Miyazaki et al. [23] directly compared diets with and without selenium but found a significant deiodinase response in the fetal brain instead of a full thyroid-gland phenotype; and Kotyzová et al. [24] primarily examined thyroid element retention under exposure to arsenic and bromine. None of these studies traced a complete sequence from baseline nutritional status through a defined molecular perturbation to hormone dysregulation and a clinically interpretable thyroid outcome, but they do support interaction at the level of tissue distribution, redox biology, and thyroid-related enzyme response.

The direction of the six rescue experiments was more consistent: at least one hormonal, oxidative, molecular, or histological consequence linked to exposure to cadmium, Cr(VI), lead, or nickel was attenuated by selenium-containing treatment. Because the thyroid has a high level of selenoprotein activity, deiodinases control the activation and inactivation of hormones, and glutathione peroxidases and thioredoxin reductases help control the peroxide produced during hormone synthesis, this pattern makes biological sense [3,35]. However, consistency of dietary effect modification is not the same as consistency of attenuation across different rescue models. Chromium and lead models employed selenium nanoparticles; the cadmium, chromium, and nickel investigations combined selenium with zinc; and the cadmium mouse research mixed selenium with myo-inositol. The distribution and reactivity of nanoparticle formulations differ from those of dietary selenium species and combination treatments, making it impossible to confidently attribute benefits to selenium alone.

Translation is further limited by dose, route, and timing. Parenteral toxicant exposure, high toxicant dosages, simultaneous co-treatment, or brief experimental intervals were used in a few investigations. These circumstances are helpful in showing whether a pathway can be affected, but they are very different from long-term low-dose exposure via food, drinking water, the air, or work situations. When extrapolating experimental results, the American Thyroid Association’s recommendations for rodent and cell research highlight the significance of species-specific thyroid physiology, hormone kinetics, assay design, and model interpretation [36]. Therefore, rather than being proof that regular supplementation will prevent disease in humans, restoration of T3, T4, TSH, antioxidant markers, or follicular shape in a rescue model should be characterized as mechanistic or therapeutic modifiability.

The risk of considering general oxidative-stress normalization as thyroid-specific evidence is also demonstrated by experimental literature. Because thyroid hormone synthesis necessitates controlled hydrogen peroxide formation and because several metals can disrupt redox balance, oxidative stress is mechanistically significant. However, without thyroid-localized measures, hormone changes, gland histology, iodide transport, TPO activity, or deiodinase-related endpoints, systemic alterations in lipid peroxidation, glutathione, or antioxidant enzymes are insufficient to demonstrate a thyroid route. No experiment systematically integrated iodide uptake, organification, redox state, deiodination, circulating hormones, pituitary feedback, and structural outcomes under deficiency, adequacy, and excess conditions. However, the strongest studies in this review included more than one of these thyroid-specific domains.

One major research gap is the lack of such a factorial experiment. The most informative future design would evaluate a chronologically ordered panel of upstream and downstream thyroid events, including metal-only and micronutrient-only controls, and expose animals to an ecologically relevant metal dose throughout well-characterized iodine and selenium states. Interpretability would be significantly enhanced by randomization, blinded outcome evaluation, predetermined primary outcomes, analytical confirmation of food composition, and characterization of baseline micronutrient status. Experimental rescue should be used to select pathways and biomarkers rather than to support supplementing recommendations until such studies are available.

### 4.4. Metal-Specific and AOP-Informed Interpretation

As shown in Figure 2, a coherent modifier model must continue to be pathway- and metal-specific. Reduced iodide absorption decreased TPO-dependent organification, thyroidal redox imbalance, and altered thyroid-hormone metabolism are the four interrelated mechanistic domains that comprise the presented evidence. Reduced hormone synthesis, altered circulating or tissue T4/T3 availability, disrupted HPT-axis feedback, and follicular structural change are all possible outcomes of these disruptions. A single universal molecular beginning event-to-adverse outcome pathway for all metals is not supported by the evidence, though. While laboratory research more frequently evaluated oxidative stress and histopathology, most human investigations tested circulating hormones. It was rare to assess NIS-mediated uptake, TPO activity, thyroidal deiodinases, or sequential HPT-axis responses directly.

This limitation is consistent with the wider thyroid disruption literature. According to Ankley and Edwards (2018) [10], AOPs are designed to arrange causal biological knowledge from a molecular initiating event through key events and key-event relationships to an adverse outcome. However, each relationship’s level of confidence is determined by biological plausibility, empirical support, dose–response concordance, temporal concordance, and applicability domain. According to Noyes et al. (2019) [4] and Haigis et al. (2023) [11], not all pathways in thyroid-related AOP networks are equally relevant to humans, and they share early events and diverge downstream effects across species. Therefore, rather than being a recently verified AOP, the paradigm employed here is AOP-informed. It places iodine and selenium as possible regulators of route advancement rather than as universal molecular initiating events and separates directly observed events from externally supported mechanistic linkages. Recent research converting thyroid-related AOPs into epidemiologically viable impact biomarkers is consistent with this conclusion [12].

Reduced availability of selenium for selenoprotein synthesis, binding and redistribution of mercury, and inhibition of selenium-dependent enzymes are the most likely selenium-related processes for mercury. A close but complicated link between mercury and selenium that varies by mercury species, selenium form, dose, tissue, and time is supported by experimental and occupational literature [37]. The lack of a consistent protective effect in human investigations could be explained by this intricacy. The toxicokinetic conditions of total mercury, cord-blood mercury, erythrocyte mercury, and occupational metallic-mercury exposure are not comparable. Similar to this, iodine-related alteration of a mercury association may function through changed hormone synthesis or thyroid substrate reserve; however, the current studies were unable to pinpoint the interaction to a particular AOP node since they did not detect NIS or TPO activity.

The species and source of arsenic are crucial for interpretation. While arsenate or arsenite were utilized in the experimental susceptibility tests, organic arsenic compounds were the main component of the seafood therapies. The absorption, metabolism, elimination, and toxicological relevance of organic seafood arsenicals, inorganic arsenic, and their metabolites vary [34]. Although selenium may have an impact on the distribution, methylation, excretion, or redox effects of arsenic, results from short-term seafood consumption cannot be immediately applied to long-term exposure to inorganic arsenic in drinking water. Therefore, anytime the suggested mechanism is species-dependent, future human investigations should detect arsenic species rather than total arsenic.

The experimental data for Cr(VI) is in line with oxidative and cellular thyroid damage that can be lessened by therapy with selenium. However, the recent rescue trials could not demonstrate that selenium status impacts risk at lower chronic dosages and instead relied on acute or parenteral exposure. Without speciation, total chromium in blood or urine should not be regarded as a Cr(VI)-specific biomarker. Thyroid buildup altered gland weight or shape, hormonal disruption, and partial attenuation by selenium-containing therapies are all supported by the literature on cadmium. It is important to avoid attributing combination effects to selenium alone because the most complete normalization frequently happened with combined selenium and zinc or selenium and myo-inositol. High-dose animal rescue dominates the evidence for both lead and nickel. All thyroid endpoints and metals should not be combined into a single mechanistic conclusion because of these metal-specific variations.

Mechanistic separation is also necessary for the endpoints themselves. Thyroid-hormone ratios, TSH, total and free T4/T3, computed homeostasis indices, antibodies, ultrasonography results, histology, and molecular markers are not interchangeable indicators of a single event. While a disruption of deiodinase activity may alter T4-to-T3 conversion without causing the same pituitary reaction, a metal that interferes with iodide transport or organification may first lower hormone synthesis and raise TSH. In rodents, follicular hypertrophy or hyperplasia may indicate extended compensatory stimulation, but depending on the species and exposure environment, it may have detrimental effects on humans. Therefore, rather than measuring a large panel and building the pathway after the fact, the most instructive future research will choose endpoints that align with a predetermined mechanistic theory.

Mechanistic separation should go beyond thyroid-specific results. Iodine or selenium status and treatment have been associated with placental redox regulation, apoptotic signaling, fetal growth, structural development, and child neurodevelopment in human observational and placental-explant studies, including settings involving metal co-exposure [38,39,40,41]. These studies are not included in the core modifier evidence set because they failed to satisfy the evaluation’s three-part eligibility requirement, which required the existence of a metal exposure, an iodine or selenium variable, and a thyroid-specific endpoint. However, they suggest biologically significant pathways and outcomes that ought to be evaluated in conjunction with thyroid objectives in subsequent studies. These investigations ought to differentiate between effects mediated by changes in thyroid hormone availability and independent extra-thyroidal effects affecting the placenta or developing organs.

### 4.5. Biomonitoring, Vulnerable Windows, and Public Health Interpretation

The most notable vulnerable window in the human evidence base is pregnancy, which is also the context where temporal misalignment has the greatest impact. Throughout pregnancy, the need for iodine, thyroid-binding proteins, renal clearance, placental transfer, and maternal-fetal hormone dependency all vary. Before the embryonic thyroid fully develops, early maternal thyroid hormones are especially crucial. Therefore, established adult reference ranges should not be extended to pregnancy without proper validation, and an exposure biomarker detected after delivery should not be immediately regarded as typical of the first-trimester exposure window. In addition to careful evaluation of iodine consumption and thyroid autoimmunity, updated clinical guidelines emphasize interpretation that is sensitive to trimester and population [33].

Evaluation of the placenta as a supplementary matrix for iodine and selenium measurement is an additional concern. Placental tissue may offer information on longer-term iodine storage at the maternal–fetal interface that is less sensitive to daily variation than a single spot urine sample, as evidenced by the correlation between placental iodine concentrations and maternal determinants as well as maternal and cord-blood free T4 [42]. Placental selenium concentrations have also been linked to fetal morphological outcomes, and first-trimester placental-explant studies demonstrate that selenium and iodine can affect oxidative stress, DNA damage, and apoptosis [40,41]. Placental measures, however, are not yet a reliable substitute for serial maternal biomarkers. Placental tissue can indicate maternal nutritional status as well as placental transport, metabolism, and disease, but it cannot resolve trimester-specific variation because it is typically only collected during delivery. Therefore, in addition to recurrent urine iodine tests, circulating and functional selenium biomarkers, speciated metal assessments, maternal and cord thyroid indicators, and longitudinal child-health outcomes, future research should assess placental iodine and selenium.

The lack of focus on sex-specific impacts is a significant research gap. Although such stratified patterns do not, by themselves, establish effect modification, Jain and Choi’s [16] included study and more general metal-thyroid investigations have found relationships that differ between males and females [16,43]. Sex-related variations in thyroid physiology, autoimmune and hormonal sensitivity, exposure patterns, and metal toxicokinetics could all be contributing factors. Future research should include an adequate number of females and males, indicate sex as a potential modifier, present sex-stratified estimates with confidence ranges, and formally test interaction. Metal-by-nutrient-by-sex interactions should also be investigated when scientifically supported and sufficiently powered. Without a direct interaction test, it is not appropriate to interpret a statistically significant association in one sex and a non-significant association in the other as proof of heterogeneity.

Other potentially vulnerable populations remain underrepresented. Individuals with autoimmune thyroid disease, renal impairment, or pre-existing iodine deficiency may react differently to both metal and micronutrient supplements. Children and adolescents may have different thyroid reserves, growth-related hormone requirements, diets, and exposure patterns, whereas older individuals may have changed renal function, medication use, and thyroid autonomy. Despite the importance of thyroid and reproductive health in this population, women with occupational exposures are likewise underrepresented. Future human biomonitoring should look beyond convenience samples and consider sex, life stage, thyroid autoimmunity, renal function, pharmaceutical use, and nutritional environment as potential sources of variation.

When individual classification is necessary, repeated spot samples, 24 h excretion, or verified consumption data should be used for iodine assessment. Dosage, formulation, adherence, and recent iodine-rich foods or medicinal exposures should all be documented when using supplements. When possible, total serum or plasma selenium should be combined with functional or transport markers such as glutathione peroxidase activity or selenoprotein P. Selenium assessment should also align with the biological window of interest. The response of selenium biomarkers to consumption might plateau at different levels, and they are not interchangeable [7]. Additionally, there is a non-linear relationship between selenium consumption and health; supplementation is most likely to change biology when baseline status is inadequate, and both low and high status can be harmful [44].

Iodine follows the same rule. Thyroid physiology can be disrupted by both excess and shortage, and those who are vulnerable may experience either hyperthyroidism or hypothyroidism with an excessive iodine load [45]. As a result, neither a high selenium biomarker nor an iodine supplement should be taken to indicate a favorable exposure category. Clinical or nutritionally justified classifications should be accompanied by continuous and non-linear analysis, and the pertinent reference distribution should be supplied. Repeat measurements, source and timing, analytical detection limits, and speciation—where toxicological interpretation depends on chemical form should all be considered when evaluating metals. Assay and reference-interval data, medication, disease, antibodies, and, if applicable, gestational timing should all be included in the thyroid assessment.

The evidence does not support iodine or selenium administration as a method for preventing metal-related thyroid dysfunction. Animal rescue doses or formulations cannot be immediately translated into dietary recommendations, and no included human trial showed prevention of clinically confirmed thyroid illness. This is especially crucial for iodine, whose excess can cause dysfunction on its own, and selenium, which has a relatively small margin between insufficient and excessive intake. Instead, then empirically supplementing populations exposed to metals, greater exposure control and nutritional susceptibility characterization are the proper public health response. Toxicology rescue studies should not be used as a basis for correcting proven deficiencies; instead, recognized clinical or public health guidelines should be followed.

### 4.6. Benefits, Limitations, and Research Priorities

This review’s modifier-centered analytical logic is its main strength. It does not combine assessments of human interactions with experimental rescue, nor does it equate measurements of iodine or selenium with proof of alteration. The distinct assessment of biomarker validity, temporal alignment, metal speciation, interaction scale, dosage relevance, and external validity lowers the possibility of overinterpretation, and the explicit H1–H4 and E1–E3 framework makes the inferential role of each study transparent. Without asserting that all metals function via the same beginning event or that every connection has been empirically shown, the AOP-informed synthesis progressively links diverse thyroid endpoints.

There are still a few limitations. Although the procedure for identifying evidence was organized and repeatable, it was not a multi-database, prospectively registered, duplicate-screened systematic review. Therefore, it is possible that eligible non-English or poorly indexed reports were overlooked. Quantitative synthesis was not possible due to the paucity of direct human modifier studies, and interaction estimates were frequently inaccurate or not fully reported. Cross-sectional designs, single-time-point status assessments, residual confounding, multiple testing, and inadequate exposure characterization were all risks associated with human studies. High dosages, parenteral methods, nanoparticles, combination therapies, and scant reporting of randomization or blinding were common features of experimental trials. The apparent constancy of protection is susceptible to publication bias because positive rescue results may potentially be publicized more frequently.

These limitations create a unique study agenda. A prospective, adequately powered biomonitoring study that consistently measures pertinent metal species and iodine and selenium status over a biologically meaningful window, prespecifies the modifier hypothesis and interaction scale, and aligns thyroid endpoints with the suggested mechanism is the highest-priority human design. In recruitment, power calculations, and statistical analysis, sex should be considered prospectively. Instead of drawing conclusions from subgroup-specific statistical significance, sex-stratified estimates and explicit interaction tests should be presented.

Micronutrient, metal, and thyroid measurements in pregnancy cohorts should be aligned over the same gestational periods, and reference intervals should be trimester- and population-specific. Placental iodine and selenium concentrations taken during delivery should be viewed as complementing tissue-integrated measurements rather than substitutes for repeated maternal urine, circulatory, or functional indicators. It would be possible to ascertain whether reported effects are mediated by separate placental and developmental pathways or by maternal or fetal thyroid disturbance by monitoring placental, birth, and child development outcomes.

Experimental studies should prioritize factorial deficiency–adequacy–excess designs at environmentally relevant metal levels, as well as examine a temporally ordered set of AOP-aligned outcomes. Together, these approaches would explain whether nutritional state influences susceptibility, identify the biological range in which alteration occurs, and differentiate physiological susceptibility from pharmacological or therapeutic relief effects.

## 5. Conclusions

Although there is currently inadequate and inconsistent human evidence to prove a repeatable protective or detrimental effect, iodine and selenium are biologically plausible modifiers of metal-associated thyroid dysfunction. Although pharmacological rescue, rather than nutritionally realistic susceptibility designs, dominates the research, experimental investigations demonstrate that selenium-containing treatments can mitigate metal-induced thyroid damage. Therefore, the conclusion that can be supported by science is conditional—while nutritional status may contribute to heterogeneity in metal-thyroid associations, neither iodine nor selenium can currently be categorized as uniformly protective or harmful, and supplementation cannot be advised as a preventive strategy for populations exposed to metals. Prospective, adequately powered human biomonitoring with repeated iodine assessment, functional selenium characterization, metal speciation, vulnerable-window sampling, predetermined sex-specific analyzes, and thyroid-specific, AOP-aligned endpoints is the top priority. Complementary placental iodine and selenium measures, as well as longitudinal developmental outcomes, should be addressed during pregnancy to differentiate thyroid-mediated effects from independent placental or extra-thyroidal routes.

## Figures and Tables

**Table 1:** Author-developed analytical framework for evaluating modifier-evidence design and interpretive significance. Categories describe evidentiary directness and do not replace risk-of-bias or certainty assessment.

Category	Operational Definition	Interpretive Role	Principal Limitations
**H1**	Formal human effect modification: interaction or product term, response-surface interaction, equivalent model-based estimate with uncertainty, or a formal heterogeneity test.	Most direct human evidence that the metal-thyroid association varies according to iodine or selenium.	Interaction-scale dependence, low power, multiplicity, residual confounding, and post hoc model selection.
**H2**	Human stratified analysis: metal-thyroid estimates reported across iodine or selenium strata without a formal interaction or heterogeneity test.	Suggestive evidence that may identify potentially susceptible groups but does not establish modification.	Data-driven cut-points, small subgroups, selective reporting, and inference from differences in statistical significance.
**H3**	Joint-exposure or mixture analysis in which the contribution, joint association, or interaction of iodine or selenium is interpretable.	Information on co-exposure patterns; direct modifier evidence only when interaction or non-additivity is explicitly represented.	Model dependence, correlated exposures, instability, and limited clinical interpretability.
**H4**	Iodine or selenium measured descriptively or used for adjustment, without an interaction, stratified effect, or interpretable joint association.	Contextual measurement only; not evidence of effect modification.	Risk of misclassification as susceptibility evidence and inappropriate causal-role assignment.
**E1**	Factorial nutritional-status experiment crossing metal exposure with controlled iodine or selenium deficiency, adequacy, excess, or multiple status levels.	Most direct experimental test of nutritional susceptibility.	Species differences, artificial status states, high toxicant doses, and limited correspondence with human exposure.
**E2**	Supplementation within a physiologically interpretable range, with appropriate metal-only and micronutrient-only controls and, where possible, baseline-status characterization.	Supportive intervention evidence within a nutritionally relevant range.	Supplementation is not equivalent to baseline status; absorption, timing, and dose comparability may be uncertain.
**E3**	High-dose, therapeutic, parenteral, post-exposure, combined, or nanoparticle intervention used to prevent or attenuate metal toxicity.	Mechanistic or therapeutic rescue evidence; not equivalent to baseline susceptibility or population prevention.	Pharmacological dosing, formulation kinetics, co-interventions, and limited translation to habitual intake.

E1–E3, experimental modifier-vidence categories; H1–H4, human modifier-evidence categories.

**Table 4:** Cross-stream synthesis of experimental and human modifier data.

Question	Human Evidence	Experimental Evidence	Evidence-Constrained Conclusion
Does iodine modify metal-thyroid effects?	One suggestive Hg-by-supplement finding, one isolated Sr-by-iodine interaction, and one broad null mixture analysis.	Limited element-distribution evidence; no comprehensive deficiency–adequacy–excess thyroid study.	Plausible but not reproducible; direction and clinically relevant status range are unresolved.
Does selenium modify metal-thyroid effects?	Small intervention suggests Se-As interaction; larger pregnancy and mixture studies do not show consistent modification.	Repeated rescue across Cd, Cr(VI), Pb, Ni, and As; only a minority are factorial status studies.	Mechanistic influence is likely; baseline human susceptibility and effective nutritional range remain unproven.
Are findings clinically translatable?	No study demonstrates prevention of diagnosed thyroid disease by iodine or selenium in metal-exposed people.	High doses, parenteral routes, nanoparticles, and co-treatments dominate.	No supplementation recommendation is justified.
What is the main methodological failure?	Single-time-point status measures, supplement proxies, poor temporal alignment, low interaction power, multiplicity, and lack of speciation.	Weak nutritional-status characterization, limited blinding/randomization reporting, unrealistic doses, and generic oxidative-stress interpretation.	Future studies must align biomarker window, metal species, thyroid mechanism, and interaction hypothesis.

As, arsenic; Cd, cadmium; Cr(VI), hexavalent chromium; Hg, mercury; Ni, nickel; Pb, lead; Se, selenium; Sr, strontium.

## Data Availability

No new data were created or analyzed in this study.

## Abbreviations

**AO**	Adverse outcome
**AOP**	Adverse outcome pathway
**As**	Arsenic
**BKMR**	Bayesian kernel machine regression
**Cd**	Cadmium
**CI**	Confidence interval
**Cr(VI)**	Hexavalent chromium
**DIO1-3**	Iodothyronine deiodinases 1-3
**E1-E3**	Experimental modifier-evidence categories
**fT3**	Free triiodothyronine
**fT4**	Free thyroxine
**GPX**	Glutathione peroxidase
**H1-H4**	Human modifier-evidence categories
**HBM**	Human biomonitoring
**Hg**	Mercury
**HPT axis**	Hypothalamic–pituitary–thyroid axis
**KE**	Key event
**KER**	Key-event relationship
**MeHg**	Methylmercury
**MIE**	Molecular initiating event
**NIS**	Sodium iodide symporter
**Pb**	Lead
**Se**	Selenium
**SPINA-GD**	Calculated peripheral deiodinase activity
**SPINA-GT**	Calculated thyroid secretory capacity
**Sr**	Strontium
**T3**	Triiodothyronine
**T4**	Thyroxine
**Tg**	Thyroglobulin
**TH**	Thyroid hormone
**THSD**	Thyroid hormone system disruption
**TPO**	Thyroid peroxidase
**TSH**	Thyroid-stimulating hormone
**UIC**	Urinary iodine concentration
